# Advancements and Limitations in 3D Printing Materials and Technologies: A Critical Review

**DOI:** 10.3390/polym15112519

**Published:** 2023-05-30

**Authors:** Syed Fouzan Iftekar, Abdul Aabid, Adibah Amir, Muneer Baig

**Affiliations:** 1 Department of Manufacturing and Materials Engineering, Faculty of Engineering, International Islamic University Malaysia, P.O. Box 10, Kuala Lumpur 50725, Malaysia; syedfouzan.iftekar@live.iium.edu.my (S.F.I.); adibah@iium.edu.my (A.A.); 2 Department of Engineering Management, College of Engineering, Prince Sultan University, P.O. Box 66833, Riyadh 11586, Saudi Arabia; mbaig@psu.edu.sa

**Keywords:** additive manufacturing, 3D printing, engineering applications, materials

## Abstract

3D printing has revolutionized various industries by enabling the production of complex designs and shapes. Recently, the potential of new materials in 3D printing has led to an exponential increase in the technology’s applications. However, despite these advancements, the technology still faces significant challenges, including high costs, low printing speeds, limited part sizes, and strength. This paper critically reviews the recent trends in 3D printing technology, with a particular focus on the materials and their applications in the manufacturing industry. The paper highlights the need for further development of 3D printing technology to overcome its limitations. It also summarizes the research conducted by experts in this field, including their focuses, techniques, and limitations. By providing a comprehensive overview of the recent trends in 3D printing, this review aims to provide valuable insights into the technology’s prospects.

## 1. Introduction

Three-dimensional printing technology has experienced unprecedented growth and is revolutionizing the manufacturing industry. This flexible technology provides the advantages of customization, prototyping, various fabrication techniques, and complex geometries at a low cost in a short timeframe. Additive manufacturing technology has come a long way since its inception when Chuck Hull, co-founder of 3D Systems, developed the first 3D printer in 1983 [1]. In the following years, there was a growing interest in this technology, and it became more affordable and accessible. In the late 1990s and early 2000s, the main focus shifted to new materials and uses, and additive manufacturing technology became more widely used in sectors such as aviation, healthcare, and automation. Today, 3D printing technology is in high demand for the way it can create complex structures with high precision and accuracy. Additionally, new techniques such as bioprinting and 4D printing have opened new possibilities in the field of medicine [2]. Metals, thermoplastics, hydrogels, extracellular matrix materials, ceramics, fiber-reinforced composites, polymers, concrete materials, and even shape memory alloys known as smart materials can be 3D printed easily because the development in additive manufacturing is at its peak and has eliminated numerous issues [3]. Moreover, this technology has fortunately introduced a new age of mass customization, where consumers have greater choices for the final product, according to their specifications. Simultaneously, 3D printing facilities can be situated nearer to the customer or even at home for personal purposes, allowing for a more adaptable and flexible manufacturing process as well as higher levels of quality control. Furthermore, the use of 3D printing technology has considerably reduced the need for worldwide transportation, saving both energy and time.

In addition to its various applications, 3D printing has revolutionized various fields of medicine, including orthopedics, surgery, and even human organs. It has enabled the production of precise surgical guides, patient-specific implants, prosthetics that can be tailor-made to fit the individual’s unique anatomy, three-dimensional tissues, and even entire functional organs and organisms [4]. Technology has shown great potential in addressing the growing need for organ transplantation, as it allows for the creation of customized, patient-specific replacement organs. Apart from medicine, three-dimensional printing has a wide variety of applications in almost every sector imaginable. This versatile technology can be used to produce goods from fashion items, food items, and toys to complex parts for aircraft and even entire rocket bodies and engines [5].

The main objective of this paper was to explore the latest trends in research and development on 3D printing as well as its advantages and limitations. Another objective was to highlight and focus on the three major types of materials used in 3D printing technology as well as two major applications for 3D printing technology.

Section 2 of this paper focuses on the types of 3D printing technology. Section 3 provides an overview of the implementation of popular materials in additive manufacturing. In Section 4, other important 3D printing materials are highlighted in order to cover all the types of 3D printing materials in this review. Section 5 discusses the expanded 3D printing applications, considering aerospace/defense, medical applications, and others.

This paper discusses the most common materials utilized in 3D printing methods, and Section 3 highlights and summarizes their important applications. The significant contribution of this paper is provided in Section 4, which critically reviews the 3D printing methods based on their materials and applications. Additionally, Section 5 offers an in-depth review of the most significant recent advancements in 3D printing technology, with a focus on metal 3D printing and fiber-reinforced composites. These two topics are highlighted since they are highly relevant to the major applications in numerous industries. Due to their great strength, high durability, and tolerance to high temperatures, metals and fiber-reinforced composites are very important. Finally, Section 6 concludes this review.

## 2. Types of 3D Printing Technology

This section provides a brief review of the various 3D printing techniques, focusing on their material systems, development, and applications. These types of 3D printing technologies offer unique capabilities that serve a wide range of businesses and applications.

Stereolithography (SLA): In the field of 3D printing, stereolithography (SLA) uses a UV laser to solidify liquid photopolymer resin layer by layer, producing solid objects with excellent surface polish and high resolution. It operates particularly well for designing complex prototypes and small-batch productions. Significant advancements have been made in stereolithography (SLA), which now offers greater accuracy, quicker print times, and a wider variety of materials. The development of resin compositions has led to the creation of biocompatible resins for use in medical applications. As a result, SLA is widely used in many different industries to create elaborate models, fine jewelry, detailed prototypes, dental and medical equipment, as well as custom parts [6].

Digital light processing (DLP): Similar to stereolithography (SLA), digital light processing (DLP) uses photopolymer resin and ultraviolet light. However, instead of a laser, it uses a digital light projector, which makes it possible to simultaneously cure a complete layer. Digital light processing (DLP) technology prints more quickly than stereolithography (SLA), although its resolution may be slightly poorer. Research on digital light processing (DLP) technology has mostly focused on increasing the material possibilities, decreasing the layer thickness, and enhancing the resolution. Rapid prototyping, jewelry casting, dental applications, and the creation of personalized consumer goods are all areas in which this technique is used [7].

Fused deposition modeling (FDM): One of the most used 3D printing procedures is melt extrusion, commonly referred to as fused deposition modeling (FDM). To build the desired object, multiple layers of hot thermoplastic filament are melted and extruded through a heated nozzle. Various materials, such as polylactic acid (PLA), metal–polymer composites, fiber-reinforced polymer composites, ceramic materials, and many different kinds of materials, can be produced using this technique. A large variety of filaments with various properties, including strength, flexibility, temperature resistance, and conductivity, is now available thanks to the development of fused deposition modeling (FDM) technology concentrated on printer design, extruder technologies, and material possibilities. Due to its adaptability, FDM can be used for quick prototyping, functional components, tooling, architectural models, and instructional applications even in the aerospace and healthcare sectors [8].

Laser ablation is the process of selectively removing material from a solid block or powder bed and molding it into the desired shape using a high-power laser beam. This technology is frequently used in the aerospace sector for manufacturing complex metal parts. Laser ablation techniques are becoming more advanced, which has resulted in greater precision and faster processing speeds. The current research primarily focuses on optimizing the laser settings and mastering the material removal techniques [9].

Multiphoton polymerization is a technique that uses high-intensity laser beams to polymerize a liquid resin, which is often aided by a photosensitive initiator. This technology allows for precise control during the curing process and is widely used in microfabrication and biological applications. Sub-micron resolutions and enhanced material characteristics have emerged from the advancements in the multiphoton polymerization processes. Researchers are continuing to investigate additional photosensitive materials and functional additives in order to broaden the technology’s potential applications. It is now used to create complicated structures, micro-optics, tissue engineering scaffolds, and other high-precision manufacturing purposes [10].

## 3. Implications of Popular Materials in Additive Manufacturing

### 3.1. Polymers

Polymers are the materials that are frequently utilized in 3D printing due to their versatility, affordability, and ease of use. They are composed of materials that can be melted and extruded into different shapes, such as plastics, resins, and rubber-like substances [11]. Fused deposition modeling (FDM) technology is the most popular method for polymer-based 3D printing [12]. There are several types of polymers used in 3D printing, each with its own unique characteristics and applications.

One of the most popular polymer materials is polylactic acid (PLA); the material that rules the world of desktop 3D printing. The applications of PLA in 3D printing include prototyping, models, DIY projects, artistic objects, household items, low-wear toys, packaging, and biomedical applications. It offers advantages such as simple printing, a wide variety of colors and patterns, and being “biodegradable.” It also has some drawbacks, such as being brittle and having weak mechanical properties. The most crucial characteristic of PLA (polylactic acid) material for 3D printing is its widespread use in FDM technology due to its low melting point, lack of toxicity, lack of irritation, and biocompatibility [13].

Another popular polymer for 3D printing is ABS, or acrylonitrile butadiene styrene. ABS is ideal for a variety of applications, including phone covers, high-wear toys, tool handles, automotive trim components, and electrical enclosures due to its good mechanical qualities, such as its impact, heat, chemical, and abrasion resistance. ABS must be printed on a heated print bed with bed glue since it is prone to warping [14].

An increasing popular material for 3D printing is polyethylene terephthalate glycol (PETG), a variation of polyethylene terephthalate (PET). PETG is appropriate for applications, including mechanical parts, printer parts, and protective components since it is flexible, strong, and simple to print. However, polyethylene terephthalate glycol (PETG) is prone to dampness and is easily scratched [15].

Flexible polymers used in 3D printing include thermoplastic elastomers (TPE), thermoplastic polyurethane (TPU), and thermoplastic copolyester (TPC). These materials are extremely flexible and durable, and they have rubber-like qualities. They are frequently utilized in products such as automotive components, home appliances, and medical supplies that require parts that can bend or compress. TPE, TPU, and TPC can be challenging to print and consider under certain printing conditions [16].

A common polymer used in powder bed fusion 3D printing is nylon, sometimes referred to as polyamide (PA). Its high levels of strength, flexibility, and durability make it ideal for applications that call for durable parts, such as tools, working prototypes, or mechanical components. However, Mohd Radzuan et al. 2023 [17] explained that using nylon in 3D printing with higher filler loadings can lead to issues such as delamination and poor adhesion. However, a post-drying process and optimized printing parameters can improve the mechanical properties, resulting in stronger samples.

One of the toughest and most resilient polymers utilized in 3D printing is polycarbonate (PC). It can withstand temperatures up to 110 °C and is resistant to heat and physical damage. Polycarbonate is frequently employed in applications such as electrical, mechanical, or automotive components, where strength and toughness are essential. The layer thickness and infill density parameters for 3D printing have a big impact on the performance of the polycarbonate pieces that were manufactured utilizing the material extrusion technique. A greater compression strength was achieved with a lower layer thickness and a higher infill density, but less energy was used with thicker layers [18].

High-impact polystyrene (HIPS) is a copolymer that combines the tensile strength of rubber and the hardness of polystyrene. It is frequently used in protective packaging and containers and, when combined with ABS, as a support material in 3D printing. HIPS has the advantage of being a reliable 3D printer filament and an appropriate support material. However, it must be post-processed to remove the supports and is only compatible with ABS. Hanitio et al. 2023 [19] looked into the effects of recycling high-impact polystyrene from electronic trash on its physical and mechanical characteristics and discovered that all of the recycled components of high-impact polystyrene had good mechanical properties and printability. Thus, even electronic waste may be used to 3D print high-impact polystyrene.

Water-soluble polyvinyl alcohol (PVA) is frequently used as a support material in intricate 3D printing with overhangs. PVA filament is an excellent support material, although it may be tricky to work with and is moisture sensitive. The rise of polyvinyl alcohol in 3D/4D printing is due to its appropriate flowability, biodegradability, and cost effectiveness, among other qualities [20].

**Figure 1:**
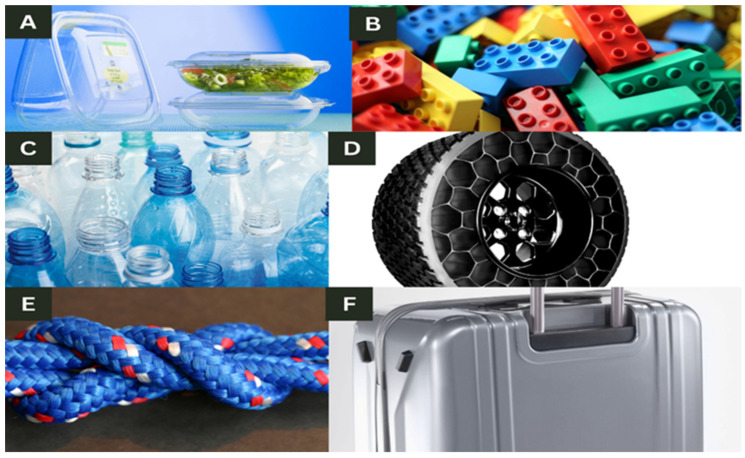
Different types of polymer materials derived from (**A**) polylactic acid (PLA)—food containers; (**B**) acrylonitrile butadiene styrene (ABS)—Lego blocks; (**C**) polyethylene terephthalate (PET)—water bottles; (**D**) thermoplastic elastomers (TPE)—airless tires; (**E**) polyamide (PA) or nylon—rope; (**F**) polycarbonate (PC)—rolling suitcase.

Acrylonitrile styrene acrylate (ASA) is a material that can withstand environmental conditions and is ideal for practical uses, particularly in the automobile sector. Due to its superior UV resistance, thermal stability, and durability, ASA filament is perfect for a variety of applications, such as sections exposed to sunshine and severe settings because it can survive external conditions without significantly degrading. Magri et al. 2022 [21] investigated the effects of the printing parameters on the tensile behaviors of 3D-printed acrylonitrile styrene acrylate (ASA) materials in the Z direction. After the analysis, the authors discovered that the layer thickness is the most significant printing parameter on interlayer bonding, and using SEM, they further confirmed that an ASA material with the best printing parameters can improve interlayer bonding in the build direction. Figure 1 highlights the different types of polymers.

Engineering plastics and food packaging both use durable, flexible, chemically resistant, and food-safe polypropylene (PP) polymers. The strong mechanical qualities, high chemical resistance, and low friction make PP filament ideal for functional parts that need to be durable, resistant to chemicals, and wear resistant. Nematollahi et al. [22] investigated the impact of polypropylene fiber addition on the properties of geopolymers created using 3D printing for digital construction and discovered that adding fibers to the pure polypropylene improved the material’s ability to retain its shape, increased the compressive strength of the material only in the perpendicular direction, and increased the material’s ductility.

A high-precision, low-friction engineering plastic is called acetal (POM). For practical applications, it is ideal because it has good resistance to heat and chemicals. However, it requires a high print bed temperature and could have issues with the first layer adhesion. Acetal (polyoxymethylene) enhanced the likelihood of a successful print without errors, providing room for further improvement [23].

The polycarbonate ABS alloy (PC/ABS) combines the flexibility of ABS with the strength and heat resistance of polycarbonate. It is frequently used in telecommunications, electronics, and the automotive industries. Functional prototypes and small-batch end-use products are most effectively manufactured using PC/ABS materials. However, PC/ABS is moisture sensitive and demands high temperatures for the nozzle and print bed. After a tensile test, Kannan et al. 2020 [24] discovered that the polycarbonate ABS alloy material showed decreased elastic characteristics and load-carrying abilities.

**Figure 2:**
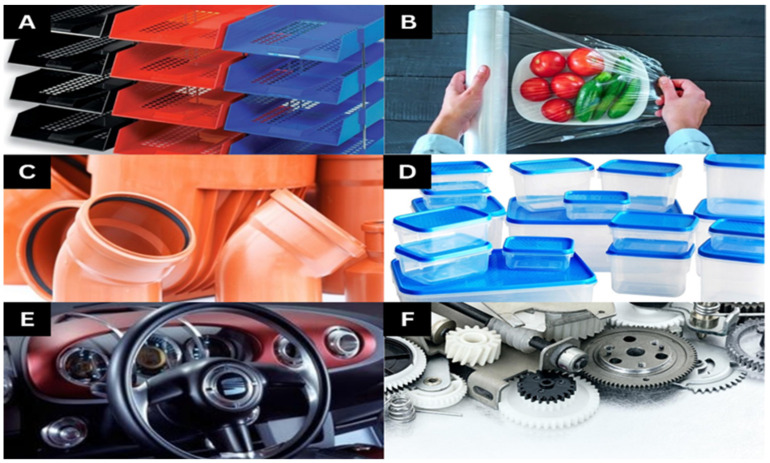
Some additional types of polymer materials derived from (**A**) high-impact polystyrene (HIPS)—stationery trays; (**B**) polyvinyl alcohol (PVA)—food wrap; (**C**) acrylonitrile styrene acrylate (ASA)—drain pipes and fittings; (**D**) polypropylene (PP)—food containers; (**E**) polycarbonate ABS alloy (PC-ABS)—glove box; (**F**) gears.

A rigid, impact-resistant, and transparent material used as a lightweight substitute for glass is polymethyl methacrylate (PMMA), often known as acrylic or plexiglass. PMMA filament is brittle, translucent, and impact resistant. However, it is rigid, can be prone to warping, and requires a high print temperature. A new 3D printing technique based on polymethyl methacrylate (PMMA), as described and tested by Polzin et al. in 2013 [25], can be utilized to create concept models, functional parts, and lost models for investment casting projects. Figure 2 shows some additional types of polymers.

### 3.2. Metals

**Figure 3:**
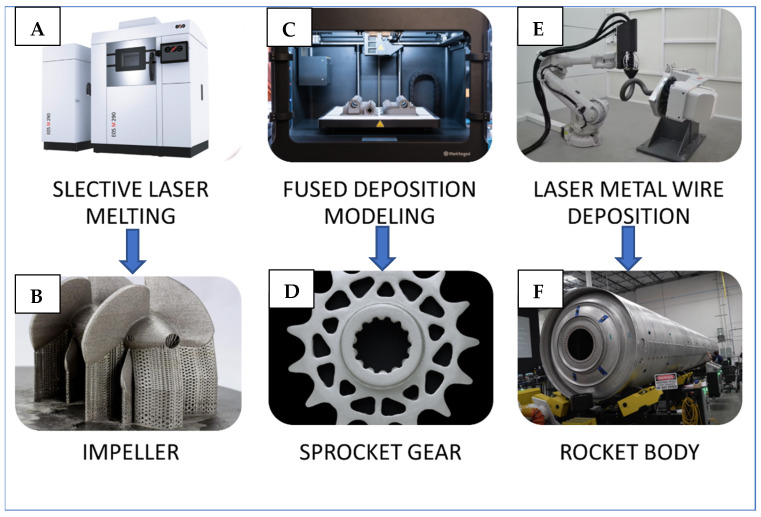
The most popular and trending metal 3D printing technologies and their applications. (**A**) Selective Laser Melting (SLM) 3D printer and (**B**) An Impeller 3D printed on Selective Laser Melting machine. (**C**) Fused Deposition Modeling (FDM) 3D printer and (**D**) A Sprocket Gear 3D printed using Fused Deposition Modeling Method. (**E**) Laser Metal Wire Deposition (LMWD) 6-dimensional robotic arm 3D printer and (**F**) A Rocket body 3D printed using this Laser Metal Wire Deposition 6-dimensional robotic arm.

A rapidly evolving field of 3D printing, 3D metal printing has gained popularity in recent years which can be seen in Figure 3. The most frequently used materials in 3D metal printing include cobalt–chromium alloy, stainless steel 316L, aluminum6061, titanium Ti6Al4V, Inconel, gold/silver, tantalum, Hastelloy, nickel chromium, tungsten alloys, and copper. These metals are widely used in the automation, healthcare, and aerospace industries [1]. Stainless steel is highly corrosion resistant and has exceptional strength when not porous [26,27]. Titanium and Ti64 are lightweight and robust metals [28]. Aluminum 7075, 4047, 6061, 2319, and 4043 are lightweight and versatile metals, while Inconel^®^ 718 and 625 are high-temperature, corrosion-resistant nickel–chromium alloys. Cobalt–chrome is biocompatible and corrosion resistant, making it useful in medical applications. Gold is highly conductive and corrosion resistant, and tantalum is used in humans to repair hard tissue defects through drug delivery systems [29]. Hastelloy is a nickel-based superalloy that is often used as a construction material in combustion chambers [30]. Tungsten is useful for radiation shields, collimators, and engine parts [31]. Since copper is a superb conductor of electricity and temperature, it is a great material for many applications such as aerospace, heating and cooling systems, induction coils, and electrical and electronic components. All these types of metals are compatible with selective laser melting (SLM) or direct metal printing (DMP) technologies, such as EOS M 400, Markforged Metal X systems, and the concept AMCM M 4K (which was used to create the first 3D-printed rocket engine) [32].

Numerous studies have explored the use of 3D printing for metal materials, each with its unique focus and limitations. Malekjafarian et al. [33] examined the buckling capacity of metal materials produced by laser 3D printing using experimental techniques but faced limitations due to the lack of a specific buckling criterion for the 3D-printed metals. Similarly, Jafari and his team studied the capillary performance analysis of metal 3D-printed wick structures for heat pipe applications using experimental techniques, but the scope of their investigation could have been expanded to include more materials [34]. Ramesh et al. [35] investigated the compression behavior of 3D-printed metal foams using both experimental and numerical approaches, but their study lacked a comparison with other types of metal foams. In the same year, Kumar et al. [36] developed a metal-plated 3D-printed electrode for the electrochemical detection of carbohydrates.

Overall, these studies provided valuable insights into the potential of 3D printing technology for metals. However, they also highlighted the challenges and limitations of these methods, such as the need for careful control of the printing parameters, the potential impact of the surface roughness and other factors on the mechanical properties, and the need for further research to confirm and extend the presented findings.

### 3.3. Fiber-Reinforced Composites

Composite materials are composed of two or more substances with combined properties that are different from the original components. They typically consist of a matrix and a reinforcement, such as carbon fiber or fiberglass [37]. This combination of materials allows composites to outperform thermoplastics while maintaining a low density. In fact, many carbon fiber layups are stronger than steel at one-tenth the weight [38]. Yang et al. [39] investigated the 3D printing process of fiber-reinforced polymer composites using several methods. Recently, several materials have been used in composite 3D printing, such as nylon-based thermoplastics and continuous fibers, including fiberglass, carbon fiber, high-temperature high-strength fiberglass, and Kevlar materials. High-strength, high-temperature fiberglass is highly resistant to heat and strength and can be used for 3D-printed welding fixtures, thermoforms, and thermoset molds [40]. Fiberglass is four times stronger and 11 times more rigid than ABS material, allowing it to 3D print industrial tools, fixtures, and work parts, whereas carbon fiber is the strongest and stiffest reinforcement fiber, with a strength-to-weight ratio nearly twice that of Aluminum6061. Kevlar can deform until the fibers begin to fail one at a time, making it ideal for applications involving multiple simultaneous motions and contacts with other parts [41].

In a further review of fiber-reinforced composite materials, Vemuganti et al. [42] examined the production of a multi-angled GFRP composite laminate with pseudo-ductile behavior. However, both studies had limitations in their models, and they suggested improving the control of the interlaminar bond strength and load transfer dynamics to enhance the ductility of 3D-printed GFRP composites. He et al. [43] analyzed the negative impact of voids in 3D-printed continuous fiber-reinforced polymer composites using optical microscopy and micro-CT, with the limitation of feasibility, while Baumann et al. [44] introduced a new method for the additive manufacturing of CFRPC using FDM. However, the results were limited due to possible variations in the material combinations and volume fraction, and further tests were necessary to evaluate other mechanical properties, such as the shear and bending strength. As a result, simulations may be required for researchers to acquire a greater understanding of the approach.

**Figure 4:**
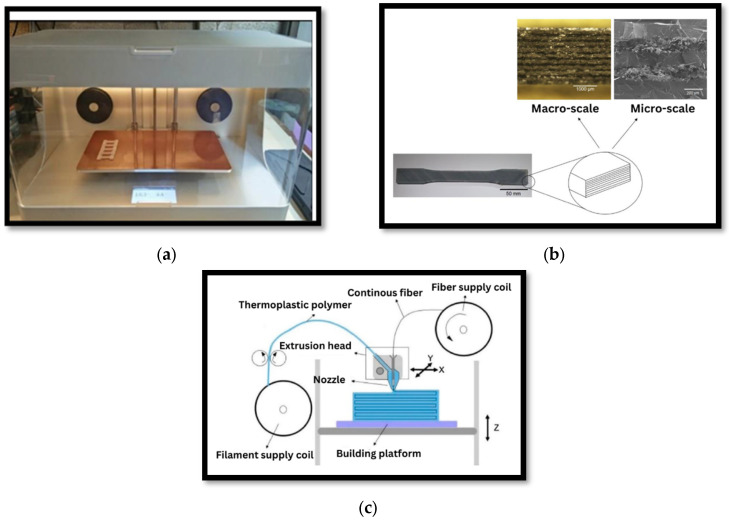
Fiber-reinforced composite 3D printing process. (**a**) actual scene, (**b**) printed specimen and (**c**) printing process [45,46]. Reprinted under the Creative Commons (CC) License (CC BY 4.0).

To advance the field of 3D printing continuous fiber-reinforced composites, future researchers should focus on improving the interlaminar bond strength control and load transfer dynamics, exploring various materials and their possible real-world applications, developing more accurate simulation models, exploring high-temperature materials, and addressing the current challenges such as the speed, resolution, energy consumption, and better hardware, software, and maintenance processes. Figure 4a shows a mark one fiber-reinforced composite 3D printer, Figure 4b illustrates a continuous fiber-reinforced composite specimen 3D printed in several layers, along with macroscopic and microscopic photographs [45], and Figure 4c shows continuous fibers and thermoplastic polymer filaments extruded continuously through a single nozzle [46].

## 4. Other Important 3D Printing Materials

One of the major advancements in 3D printing has been the development of new materials. Previously, 3D printing was restricted to plastic materials. However, a wide range of materials can now be used to produce high-quality parts and products.

### 4.1. Smart Materials

Smart materials can change their properties or behavior in reaction to environmental factors such as temperature, pressure, light, or magnetic fields. The unbelievable part is that these changes can happen within a few seconds or milliseconds. Smart materials can be used in 3D printing processes such as stereolithography (SLA) and fused deposition modeling (FDM) to create objects with shape memory capabilities. These materials can accommodate user requirements, are incredibly adaptable, and provide limitless options [47]. There are several kinds of smart materials, and each has special qualities and uses. A few illustrations are explored below.

- Shape memory alloys (SMA) are interesting metals with a fascinating ability to “remember” their original shape. Therefore, if they are distorted, they may be restored their original shape by heating or cooling. Nitinol, an alloy consisting of nickel and titanium, is a well-known shape memory material. It is included in surgical instruments and implantable medical equipment. These implants may be compressed and then heated before being inserted into the body to restore their original form and functionality.
- Ferrofluid: Ferrofluid is a substance composed of minute magnetic particles floating in a liquid. The particles align and stiffen the substance when it is exposed to a magnetic field. To accurately regulate the loudspeaker’s diaphragm’s movement, ferrofluid is frequently utilized in loudspeakers. Additionally, it could also be used to seal items.
- Magnetorheological (MR) fluids: Similar to ferrofluids, magnetorheological (MR) fluids are composed of tiny magnetizable particles. However, compared to ferrofluids, magnetorheological (MR) material particles are bigger. Brakes and adaptive damping systems frequently employ these materials.
- Electroactive polymers (EAPs): When exposed to an electrical field, EAPs, which are intelligent materials, alter their structure, size, or volume. Electroactive polymers (EAPs) are fascinating because of their remarkable flexibility, high load capacity, and quick reaction times. They can be used in soft robotics, energy harvesting technology, and artificial muscles. Electroactive polymers (EAPs) are light weight, have a low power consumption, and are compatible with various production processes, making them favorable over conventional actuators.
- Piezoelectric materials: These clever materials can convert mechanical energy into electrical energy and vice versa. They are commonly employed in sensors, actuators, transducers, and energy harvesting devices. Piezoelectric materials produce an electric charge separation when mechanical stress is applied, whereas an electric field induces mechanical deformation. While there are natural and artificial piezoelectric materials, artificial materials such as lead zirconate titanate (PZT) are commonly used due to their high sensitivity and output signals.
- Chromogenic materials: These materials have the capacity to alter their color or optical characteristics in response to a variety of external stimuli, including electric fields, heat, light, and mechanical stresses. Sunglasses with photochromic lenses, which get dark when exposed to UV light, is a well-known example. Chromogenic materials are used in security inks, temperature-sensitive paints, and smart windows, as well as a few other applications.

The fascinating and exciting world of smart materials demonstrates that materials are more than just rocks, metals, or plastics. Smart materials have numerous product advantages, from self-healing materials that automatically fix themselves when damaged to smart fabrics that can warm or cool humans when necessary. Incredible developments in smart materials will continue due to advances in materials science and technology, which will enable researchers to design smarter, more adaptive, and more sustainable solutions. Future advancements in this field are exciting, which is why we can look forward to how smart materials will alter our way of life [48].

### 4.2. Ceramic Materials

With new opportunities and applications, ceramic materials are making major advancements in the field of 3D printing. Ceramics’ high temperature resistance, hardness, and electrical insulating qualities have long been valued in conventional production. Due to their brittleness and demanding fabrication requirements, ceramics materials caused difficulties for 3D printing. Ceramics can now be explored in 3D printing due to recent developments in additive manufacturing technologies that have removed these barriers [49]. Ceramic powders or pastes are used as feedstock materials in ceramic 3D printing, sometimes referred to as ceramic additive manufacturing. The ceramic material is deposited and shaped layer by layer using a variety of processes, including selective laser melting, stereolithography, and binder jetting. The complex geometries and detailed designs that were previously challenging to produce using conventional ceramic manufacturing techniques are now possible. Additionally, ceramic 3D printing provides customization and design optimization options, enabling the fabrication of ceramic components with specialized qualities and functions. The performance, density, and strength of the printed ceramic pieces can be further improved by post-processing methods such as sintering. Although there are still difficulties, such as producing high-density pieces without flaws and accelerating production, ceramic 3D printing is getting better. 

### 4.3. Bioink Material

**Figure 5:**
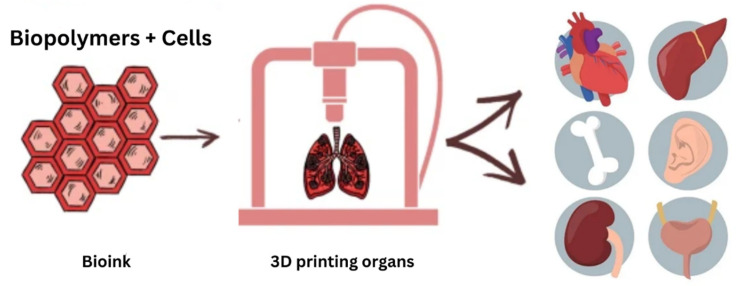
The bioprinting process.

Bioinks are materials used in 3D bioprinting to create tissues and organs. They act as a support system for living cells during printing. Hydrogels such as alginate and gelatin as well as synthetic polymers such as PCL and PLA, are common bioink materials. Cell-laden bioinks involve mixing cells with a carrier material such as a hydrogel or using cell aggregates. The application of bioink has great potential in personalized therapies with increased concentration for controlling drug releases, drug screenings for cancer treatment, studying the possible side effects, and analyzing the behavior of tumor cells, etc. [50]. Most of the time, the BI formulations are hydrogel-based since they are formed by water-rich materials that more closely reproduce the extracellular matrix (ECM) environment. The use of biocompatible and biodegradable ingredients, together with the inclusion of cells within bioink, make it possible to print customized structures or tissues with minimal healing time as well as minimal chances of implant rejection and other immune responses. At present, 3D bioprinting has enabled the in vitro production of complex tissues, including skin, cartilage, bone, lung, and heart. Figure 5 shows the bioink materials processing.

The bioengineered materials used in 3D printing have beneficial properties for human health. They are kind to our cells, support their growth and development, permit the movement of nutrients, and can safely degrade without making people ill.

## 5. 3D Printing Applications

**Figure 6:**
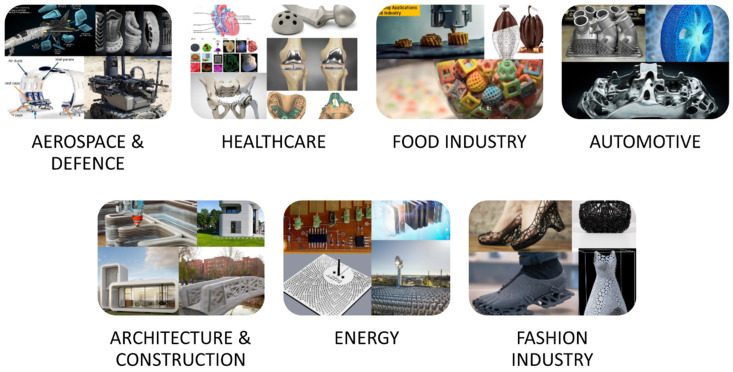
Major applications of 3D printing.

To date, 3D printing has been used in a variety of applications, ranging from consumer products to complex industrial components. Some of the major applications of 3D printing are presented in Figure 6.

### 5.1. Aerospace and Defense

The application of 3D printing technology in the aerospace and automotive industries has been widely recognized. From its inception, not only has additive manufacturing served as a rapid prototyping method for cost-effective and time-efficient product development but it has also had a profound impact on designing products, directly manufacturing individual parts, and assembling and repairing parts even in the aerospace sector. When compared to traditional manufacturing methods, AM creates stronger and lighter products with excellent mechanical properties. This technology has also been applied in the automotive sector, enabling the production of lighter car parts, components, and prototypes with faster turnaround times. Furthermore, 3D printing can also manufacture replacement and spare parts more efficiently.

NASA’s Langley Research Center and Glenn Research Center are researching various additive manufacturing techniques to improve the efficiency and strength of aircraft, engines, rocket propulsion components, and spacecraft. Langley researchers are using additive manufacturing to create parts composed of special alloys that are tougher than regular metal and fit together better. Glenn researchers are exploring the use of polymers and ceramic composites to develop gas turbine engine parts that could reduce emissions and improve efficiency. They are also using selective laser melting and electron beam freeform fabrication to create components for rocket propulsion [51]. Additionally, powder bed fusion (PBF) technology is another technique used by NASA to produce complex and expensive parts, such as the RS-25 flex joint, which enables faster production with intricate details. NASA is also developing 3D printing and additive manufacturing technologies for use in space, specifically on the International Space Station (ISS) using melt deposition modeling controlled by a software called MIS SliceR. However, there are limitations to the technology, including a limited print volume and potential inaccuracies due to the microgravity environment.

**Figure 7:**
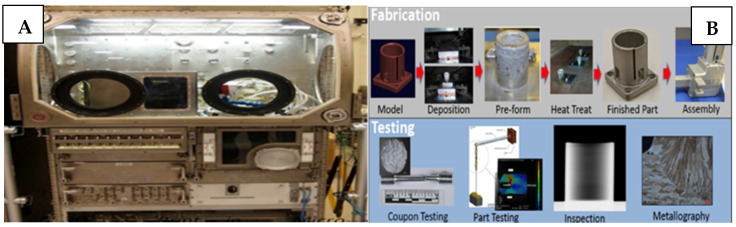
Samples of aerospace applications. (**A**) First 3D printer taken to International Space Station, (**B**) Fabrication and Testing of various parts required in Space Station [51]. Reprinted under the Creative Commons (CC) License (CC BY 4.0).

NASA is also utilizing additive manufacturing to reduce the cost of rocket engine programs by developing copper combustion chambers using powder bed fusion and selective laser melting techniques, as part of the Low-Cost Upper Stage Propulsion (LCUSP) program. Hot-fire tests have been conducted to demonstrate the efficacy of these techniques for different thrust applications. These efforts demonstrate NASA’s commitment to utilizing innovative manufacturing techniques to reduce costs and improve efficiency, further advancing the field of space exploration. Figure 7 shows the use of 3D printing to create fine internal geometries, environmental control, and life support systems, and designing tools for manufacturing and processing in space, specifically on the International Space Station (ISS). NASA’s goal was to use 3D printing technology to manufacture and analyze copper combustion chambers, and then test them by igniting fuel in conditions with no post-production work on the coolant channels inside the chambers [51,52].

In addition, researchers have studied penetrative combustion in 3D-printed rocket fuel grains [53]. Grefen et al. [54] examined 3D-printed molds for complicated solid fuel block designs utilized in hybrid rocket engines. The report also included multiple engine tests with various fuel grain shapes and advised that black carbon should be blended with solid fuel to improve performance. Deters [55] highlighted the current and potential applications of 3D printing in the aerospace and defense industries and Joshi et al. [56] conducted a review of 3D printing in aerospace and its sustainability, noting numerous challenges to overcome, such as printing patterns, irregular print flow, and porosity issues, to ensure its long-term sustainability. According to these studies, 3D printing in aerospace is still in the early stages and needs to be further developed to ensure its long-term sustainability.

### 5.2. Biomedical and Healthcare

From the creation of custom prosthetics and implants to the printing of surgical guides and organs, 3D printing technology has an enormous number of applications in the healthcare industry. Organ 3D printing has demonstrated significant progress in both animal and human models, paving the way for potential developments in transplantation and regenerative medicine. The technology has also been used to produce personalized medicine, such as customized pills with specific dosages and active ingredients. With the ability to create precise and intricate structures, it has the potential to transform the way the medical industry operates [57].

**Figure 8:**
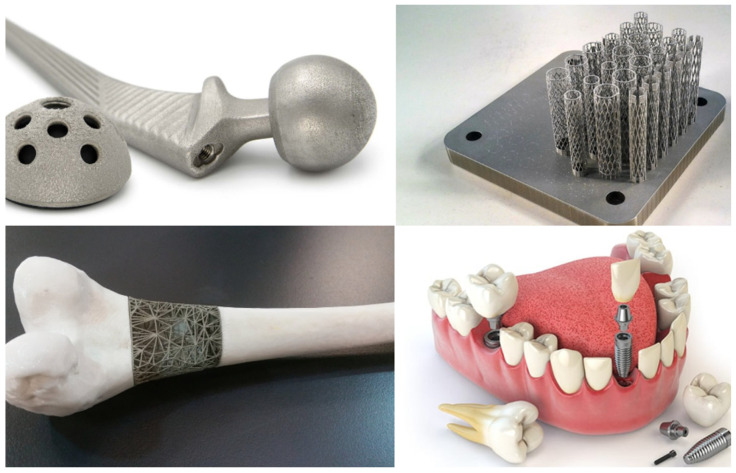
Scaffolds built of metal powders and manufactured via SLM 3D printing.

Cornelis Vlasman [58] and his team led the project, which has enormous potential for both medical and research applications. The modular body can be used for medical testing and research, notably to explore the long-term and short-term consequences of the harsh conditions encountered during space travel. In 2021, Chua et al. [59] introduced a groundbreaking idea that discussed the utilization of 3D printing technology with metallic biomaterials in the biomedical field, emphasizing the advantages and drawbacks of this approach. The review acknowledged the challenges associated with metallic biomaterials, such as the low tensile strength, poor surface roughness, and high cost. It also recognized the difficulties in achieving a high dimensional accuracy and fine structures using traditional manufacturing methods, as well as the limitations of 3D printing in terms of a lower precision and resolution compared to other additive manufacturing techniques. Despite these limitations, the authors emphasized the potential benefits of 3D printing technology for creating customized medical devices, such as stents used in angioplasty procedures, and the additive manufacturing processes that can be used to improve their performance for specific medical applications (Figure 8).

**Figure 9:**
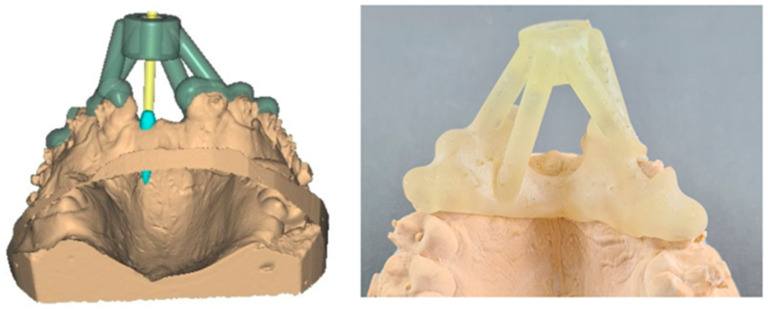
Simulated 3D-printed insertion tool composed of biopolymer material [60]. Reprinted under the Creative Commons (CC) License (CC BY 4.0).

The study conducted by Nagib et al. [60] examined the use of a polymeric 3D-printed surgical guide for non-standard mini-implant orthodontic cases. They performed an FEM simulation using the Abaqus numerical analysis software. While the guide showed promising results for improving treatment outcomes, further research with different operators is required to ensure its effectiveness in diverse scenarios for official implementation. Similarly, Zhao et al. [61] developed a new strategy for creating dental crowns that closely mimicked the multi-scale structure of natural tooth enamel. This method was more efficient and reliable than traditional methods and produced crowns with greater accuracy and strength, which could have significant implications for the field of dental care. Figure 9 shows a 3D-printed personalized surgical guide for the placement of mini-implants in front of the maxilla. It features a blue simulated topological associating domain model, and the yellow component simulates the 3D-printed insertion tool.

### 5.3. Other Applications

#### 5.3.1. Food Industry

The food industry has embraced 3D printing technology to create new and innovative food products. Overall, 3D printing allows for the creation of intricate shapes and designs that would otherwise be difficult to achieve through traditional methods. This has resulted in the development of novel and unique snacks, desserts, and even complete meals that are both aesthetically pleasing and delicious. The technology also has the potential to produce complex geometrical shapes in a shorter period, making it easier to produce healthier food products with precise control over the used ingredients [62].

#### 5.3.2. Automotive Industry

The automotive industry is using 3D printing to create lighter and stronger parts for cars, leading to improved fuel efficiency and performance. The technology also allows for the rapid prototyping and testing of new designs, minimizing the time and expenses required to launch a new product [63]. Additionally, custom, and specialized parts are being manufactured using 3D printing to maintain unique and vintage cars, offering owners a more convenient option for vehicle preservation.

#### 5.3.3. Architecture and Construction

3D printing has revolutionized the architecture and construction industries by allowing for the rapid prototyping of building designs and the creation of complex and intricate structures. Technology is also being used to create customized and unique building components, such as wall panels and tiles, which would be impossible to produce using conventional methods [64]. With the ability to create precise and intricate structures, 3D printing has the potential to change the way buildings are designed and built. New and novel construction methods are necessary to accomplish the worldwide aim of lowering carbon dioxide emissions. These technologies should not only promote green building practices but also reduce the costs of creating and managing facilities while maintaining a competitive advantage.

#### 5.3.4. Energy

Using 3D printing to produce energy conversion technologies could be a major shift. It could be a low-cost strategy that allows for the manufacturing of complicated designs and improved performance per unit of mass and volume. It can be used to create intricate and customized components for renewable energy systems, such as wind turbines and solar panels. Additionally, it is possible to reduce waste and improve efficiency in energy production by enabling the creation of precise and optimized components [65].

#### 5.3.5. Fashion Industry

Advances in 3D printing have been embraced by the fashion industry to create unique and innovative clothing and accessories. Printing 3D designs onto fabric eliminates the need for glue, and the bonding between the fabric and printing materials is primarily due to physical locking rather than chemical bonding. [66]. From light and complex parts to unique and innovative clothing and accessories, 3D printing has created new opportunities to produce customized and personalized clothing.

## 6. Advancements and Limitations

**Figure 10:**
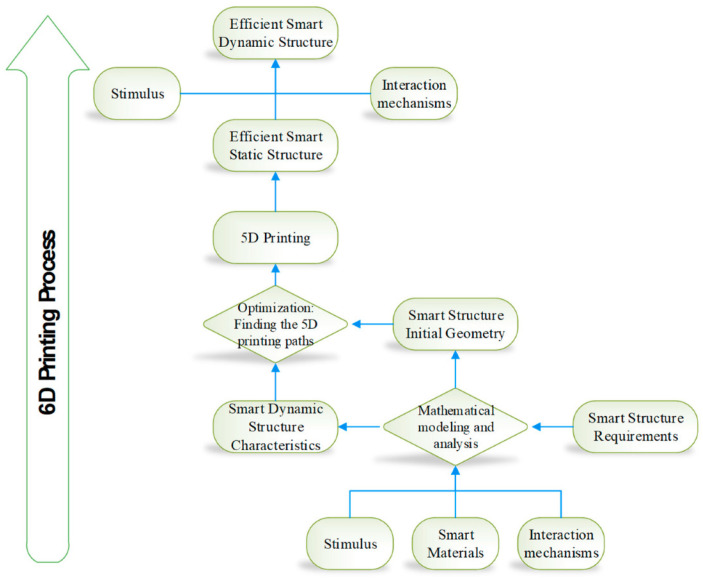
The concept of 6D printing [67]. Reprinted under the Creative Commons (CC) License (CC BY 4.0).

Georgantzinos et al. [67] explored the concept of 6D printing, which combines 4D and 5D printing techniques, with a focus on the benefits and limitations of the process, as well as the use of smart materials and their application in the additive manufacturing industry. However, these techniques require specialized equipment and materials, leading to higher setup costs and potential limitations in structural complexity and processing times. Future research could explore ways to mitigate these limitations and improve the efficiency and affordability of 6D printing (Figure 10). Meanwhile, there have been several comprehensive reviews of recent developments in 3D and 4D printing. For instance, Khoo et al. [47] presented a detailed overview of recent major advances in 4D printing, including the 3D printing of various materials and structures for engineering applications. Similarly, Deshmukh et al. [2] provided an introductory outline of 3D and 4D printing technologies and highlighted their potential applications in many kinds of domains, including biomedical engineering and aerospace.

Lee et al. [68] focused on the application of 3D printing technology to improve the design of membrane modules. Additionally, Balogun et al. [69] introduced various 3D printing techniques for fabricating module spacers and membranes. Both studies discussed recent developments around water treatment systems and how 3D printing technology has improved their efficiency. Zhang et al. [70] highlighted the most recent advancements and trends in 3D printing mesostructured materials, while Costa et al. [71] summarized the most recent developments in 2D and 3D-printed batteries.

In addition, Tay et al. [72] reviewed the most recent developments in 3D printing by examining papers from 1997 to 2016. The authors provided a comprehensive analysis of the field’s growth and development, challenges, and opportunities for future research. They also discussed the latest developments in material design and fabrication techniques, as well as the potential applications of 3D printing technology in energy storage. Recently, Munina et al. [73] examined the present state of variable refractive lens antennas, highlighting the advantages and challenges related to 3D printing and discussing the various types of 3D-printed lenses and their applications. Although the study provided a comprehensive overview of 3D-printed radar antennas, further research may be necessary to explore their full potential.

Furthermore, a company discovered a radix printable dielectric substance, which is the first UV-curable 3D printing resin developed for antennas and radio frequency (RF) lens applications. This material allows designers to use creative design with additive manufacturing to improve the performance and flexibility. Similarly, Jeong et al. [74] also developed a low-cost Doppler radar system using 3D-printed horn antennas that can effectively measure human vital signs. Although the system was validated for accuracy and has potential applications in the healthcare, automotive, military, and security industries, further research is needed to assess its scalability and durability.

Finally, some researchers have identified a new method for fabricating ceramic components that are 3D printed using pre-ceramic monomers and ultraviolet photo initiators, which shows advancement in the field. However, the study may benefit from further investigation into the mechanical and thermal characteristics of the manufactured parts to determine their suitability for various applications.

### 6.1. Summary and Limitations

In this review, a summary from each paper has been designed into a table (Table 1) considering three main factors one is the focus of their studies, next is what kind of technique they adopted and finally what are limitation from their work which can be able to fill in the future studies. With this the early researcher can get idea of finding the research gap in this field. 

### 6.2. Discussion

A discussion has been provided based on the current review of the work in each section, which was limited to the references provided in this study.

- Applications: 3D printing technology has become commonplace in a variety of industries, including healthcare, aerospace, automotive, and manufacturing. Its major applications include rapid prototyping, tooling, and end-use part production. Furthermore, 3D printing can solve societal problems, such as food scarcity and homelessness, by enabling the government to fund the production of free foods and homes for people in need. However, the development of this technology is still in its early phases, and more research is needed to explore its full potential.
- Materials: The materials used in 3D printing technology include thermoplastics, metals, ceramics, and composites. Despite the wide range of materials available, there is still a need to develop high-strength and high-temperature materials that are more affordable. The research on materials is continuously advancing, but there is still a considerable way to go.
- The direction of research: The amount of research on 3D printing technology is consistently increasing, but limitations of the research have been acknowledged. Many studies have only focused on a limited range of materials, with insufficient information, a lack of in-depth study, and a smaller number of samples, thereby reducing the overall understanding of the consistency of the experiments. More research is needed to address these limitations and explore the potential of this trending technology.
- Cost and speed: The cost and speed of 3D printing technology remain significant challenges. While the average cost of 3D printers has decreased in recent years, the cost of materials and maintenance remains high. In addition, when compared to conventional manufacturing methods, the speed of 3D printers is still slow. More experimentation and innovation will be needed to cut prices and increase the speed of 3D printing technology.
- Sustainability: The 3D printing industry is focused on sustainability by developing eco-friendly materials and adopting circular economy models. This is a crucial area of research that desperately needs to be explored further.

Overall, while 3D printing technology has advanced significantly in recent years, there are still many challenges that need further development rather than investigation. Thus, considerably more research is needed to explore the potential of this technology, develop new materials, and improve the cost and speed. Additionally, sustainable practices and the potential impact of 3D printing on society must be considered.

### 6.3. Research Gap

Based on the above studies, some potential research gaps in the field of 3D printing technology have been presented.

- Limited research on high-strength and high-temperature materials that are affordable: Although there has been significant progress in the development of new materials for 3D printing, there is still a need for more research on high-strength and high-temperature materials that are more affordable for widespread use.
- Insufficient details and clarity in research studies: Many studies in the field of 3D printing technology have been criticized for providing insufficient details and clarity in their explanations. Future research should aim to provide more detailed and transparent reporting of the research methodologies and results.
- Lack of in-depth studies and smaller sample sizes: While there has been a rapid increase in the research on 3D printing, many studies have only focused on a limited range of materials, with insufficient information, lack of in-depth studies, and smaller sample sizes. Future research should aim to conduct more comprehensive studies with larger sample sizes to improve the consistency of the experiments.
- Limited research on the environmental impact of 3D printing: While there is growing interest in the potential of 3D printing technology to enable sustainable manufacturing, there is still limited research on the environmental impact of 3D printing. Future research should focus on developing eco-friendly materials and adopting circular economy models.
- Limited research on 3D printing in medical contexts: While there is potential for 3D printing technology to be used in medical contexts to lower the cost of surgeries for patients who cannot afford the current cost chain of manufacturers, sellers, hospitals, and doctors, there is still limited research in this area. Further research is needed to explore the potential applications of 3D printing technology in healthcare.
- Limited research on the societal impact of 3D printing: While there is growing interest in the potential of 3D printing technology to address societal challenges, such as food scarcity and homelessness, there is still limited research on the societal impact of 3D printing. Future research should focus on exploring the potential of 3D printing technology to enable the production of free foods and homes for people in need, and to reduce the fear and impact of poverty.

## 7. Conclusions and Recommendations

This paper critically reviewed the recent trends in 3D printing technology, including its major applications and the materials used. It also examined the direction of research, the methods, and their associated limitations in this field. To achieve greater success in the additive manufacturing industry, further experimentation and innovation are needed to reduce costs, increase speeds, and develop high-strength and high-temperature materials that are more affordable. The ultimate goal is to generalize 3D printing technology to enable the manufacturing of essential items at home and in medical contexts, such as lowering the costs of surgeries for patients who cannot afford the current cost chain of manufacturers, sellers, hospitals, and doctors. Additionally, this technology can solve other societal challenges, such as food scarcity and homelessness, by enabling governments to fund the production of free foods and homes for people in need, thereby reducing the impact of poverty. The literature shows that there has been a rapid increase in research on 3D printing. However, the limitations of the research have been acknowledged, as there are still numerous possibilities for conducting further experiments. In some cases, researchers did not provide sufficient explanations and details, and many studies focused only on a limited range of materials, with insufficient information, a lack of in-depth study, and a smaller number of samples, thereby reducing the overall understanding of the consistency of the experiments. Additive manufacturing is expected to continue advancing and improving, but it will take some time to overcome the challenges, particularly those related to the cost and speed of 3D printing. As technology becomes more efficient, faster, and cost-effective, it will become more accessible to a wider range of users worldwide. Additionally, the industry will focus on sustainability, developing eco-friendly materials, and adopting circular economy models. Overall, the future of additive manufacturing looks promising, and it will be fascinating to witness the emergence of innovations and applications in the years to come.

## Figures and Tables

**Table 1:** Summary and limitations of recent work.

Ref.	Focus	Technic Adopted	Limitations
Malekjafarian et al. [33]	Metallic materials created using 3D printing with lasers can resist bending.	Data analysis of the metal material.	The bending behavior could have an impact on 3D-printed metal parts by limiting their strength and durability during manufacturing.
Jafari et al. [34]	Testing of 3D-printed metal filaments heat transfer pipes.	Additive manufacturing for stainless steel porous structures followed by running liquid through those pores to measure the time.	Limited sample size, and a lack of generalizability to the other materials.
Ramesh et al. [35]	How 3D-printed metal foams behave when they are compressed.	Selective laser sintering to print the metal; used both experimental and numerical methods.	Comparison of the metal foams.
Yang et al. [39]	3D Printing of fiber-reinforced polymer composites.	Concentrated on fused deposition modeling and a discrete element technique.	2D model.
Dickson et al. [41]	Carbon, Kevlar, and glass fiber-reinforced polymers.	FDM technology was used for 3D printing and a mechanical test.	Material combinations were subject to change and the volume fraction could change.
Vemuganti et al. [42]	A laminate of fiber-glass-reinforced composite from multiple angles.	3D finite element (FE) model and experiments.	The impact of the interlaminar bonding capacity controlled by 3D printing.
He et al. [43]	Impact of voids or cracks on 3D-printed continuous fiber-reinforced polymers.	Micro-CT and optical microscopy.	Root cause of the void formation during the 3D printing process.
Baumann et al. [44]	A continuous fiber-reinforced polymer.	Experimental technique.	Overprinting of the fiber strands, placement of the fiber strands through a needle.
Grefen et al. [54]	Potential of creating molds for the complicated shapes required in hybrid rocket engines.	3D printer to create different shapes of fuel blocks.	Further research and testing are required to determine whether it works well.
Nagib et al. [60]	Examining the polymeric 3D printing guides for orthopedic implants.	FE method for performing the simulation to analyze the problem.	More research was needed with different operators, to ensure the strength of the guide and that it will work in different situations.
Zhao et al. [61]	Dental crowns with a highly ordered structure that mimics the multi-scale structure of natural tooth enamel.	3D printing and direct ink writing.	Limited to different materials.
Munina et al. [73]	Application of lens antennas with a specific feature known as the “gradient refractive index”	Fused deposition modeling technique to produce the complex structure of the antenna.	Impacted the performance of the antennas at higher frequencies.
Jeong et al. [74]	A low-cost radar system to detect the vital signs of a person.	The experimental method, signal processing with electrical components, and data analysis using the MATLAB software.	Lack of detailed information about the design and fabrication process of the gradient refractive index lens antennas.
Goh et al. [75]	Characterization of mechanical properties of additively built carbon fiber and glass fire-reinforced thermoplastics.	Experimentation with fused filament fabrication (FFF) 3D printing from Stratasys.	Experimental approach.
Yamawaki et al. [76]	Fabrication and mechanical characterization of continuous carbon fiber-reinforced thermoplastics.	Mechanical characteristics were measured.	Possibility to conduct a parametric investigation and numerical simulation.
Abadi et al. [77]	Elastic characteristics of 3D-printed fiber-reinforced polymer (FRP) structures.	Method of analytical volume average stiffness (VAS) and Numerical Abaqus.	Limited to 2D models.
Yao et al. [78]	Ultimate tensile strength of polylactic acid (PLA) materials.	Experimental work specimens with different thicknesses and different angles of printing.	Could determine the angle at which the interlayer or inlayer fracture occurs.
Adumitroe et al. [79]	Composite microstructure and the performance of 3D-printed materials.	Toughened semi-interpenetrating polymer network—thermoplastic polymer bi-matrix composite FFF technology.	Could utilize high-temperature epoxy or TS polymers for high durability and mechanical strength.
Forcellese et al. [80]	3D printing of isogrid structures.	Experimental fused filament fabrication (FFF) printing and testing.	Polyamide reinforced with short carbon fibers were manufactured; only compression test.
Polyzos et al. [81]	Mechanical properties of 3D-printed materials with continuous fibers using the multi-scale analytical technique.	Experimental and multi-scale analytical technique.	The analytical method predicted the stress or strain from the addition of cross-wood laminates while considering the failure conditions and remaining hygrothermal stresses.
Zhang et al. [82]	Failure of weaved composite plates with holes under tensile and shear stresses.	The finite element software to predict and analyze the failure of 3D-printed weaved plates.	3D printing could be used for manufacturing continuous fiber-reinforced composites.
Todoroki et al. [83]	Tensile properties of composites reinforced with continuous carbon fibers using thermoplastics.	Experimental tensile test with different orientations.	Only the fiber direction was considered during the tensile test.
Dou et al. [84]	Process parameters of 3D printing continuous CFRP composites.	Experimental 3D printing and specimen test.	Enhancing the point of contact between the carbon fiber and the matrix component.
Pascual-González et al. [85]	Influencing variables in the microstructural reaction.	Experimental method.	Micromechanical behavior of the 3D-printed composite.
Sugiyama et al. [86]	Continuous fibers to 3D print composites with varying stiffnesses and fibers.	Experimental approach.	Could increase the strength and stiffness of the samples by improving the design and using an appropriate filament-cutting mechanism.
Wei et al. [87]	Arrangement of various 3D printing methods that affect the properties.	3D printing and tensile testing.	Factors such as chemical composition and microstructure were not considered.
Koh et al. [88]	Adding nano bits of fumed silica to 3D printing to make stronger steel parts.	Used the SLM technique to fabricate the specimens.	The results were based on a small number of samples, and there was no consistency in the sample outcomes.
Kogo et al. [89]	Amount of weight 3D-printed metals can hold without breaking.	Additive manufacturing and mechanical tests.	The scanning strategy utilized in the study may not have been appropriate or optimal.
Karolewska et al. [90]	Testing titanium alloy and observing its strength while the load is stable.	SLM method to print the titanium alloy.	Lack of consistency in reporting the important parameters and the influence of the factors.
Król et al. [91]	Experimenting with 3D printing maraging steel and investigating various parameters.	Selective laser melting to print the steel and examine the part porosity, microstructure, and hardness test.	The lack of information on the impact of the temperature and ageing time on the maraging steel.
Mergulhão et al. [92]	The strength and structure of cobalt–chromium (Co–Cr) alloy.	3D-printed cobalt–chromium alloy was used for selective laser melting and further tests.	The mechanical characteristics or microstructures of the samples changed between the SLM and casting techniques.

## Data Availability

Not applicable.
